# Effectiveness of metal oxide catalysts for the degradation of 1,4-dioxane†

**DOI:** 10.1039/c9ra05007h

**Published:** 2019-08-28

**Authors:** Kimberly N. Heck, Yehong Wang, Gang Wu, Feng Wang, Ah-Lim Tsai, David T. Adamson, Michael S. Wong

**Affiliations:** Department of Chemical and Biomolecular Engineering, Rice University Houston TX 77005 USA mswong@rice.edu; Dalian Institute of Chemical Physics, Chinese Academy of Sciences Dalian China; Division of Hematology, Department of Internal Medicine, University of Texas-Medical School at Houston Houston TX 77030 USA; GSI Environmental Houston TX 77098 USA; Department of Chemistry Houston TX 77005 USA; Department of Civil and Environmental Engineering Houston TX 77005 USA; Department of Materials Science and Nano Engineering Houston TX 77005 USA; Center for Nano-Enabled Water Treatment, Rice University Houston TX 77005 USA

## Abstract

1,4-dioxane, commonly used as a solvent stabilizer and industrial solvent, is an environmental contaminant and probable carcinogen. In this study, we explored the concept of using metal oxides to activate H_2_O_2_ catalytically at neutral pH in the dark for 1,4-dioxane degradation. Based on batch kinetics measurements, materials that displayed the most suitable characteristics (high 1,4-dioxane degradation activity and high H_2_O_2_ consumption efficiency) were ZrO_2_, WO_*x*_/ZrO_2_, and CuO. In contrast, materials like TiO_2_, WO_3_, and aluminosilicate zeolite Y exhibited both low 1,4-dioxane degradation and H_2_O_2_ consumption activities. Other materials (*e.g.*, Fe_2_O_3_ and CeO_2_) consumed H_2_O_2_ rapidly, however 1,4-dioxane degradation was negligible. The supported metal oxide WO_*x*_/ZrO_2_ was the most active for 1,4-dioxane degradation and had higher H_2_O_2_ consumption efficiency compared to ZrO_2_. *In situ* acetonitrile poisoning and FTIR spectroscopy results indicate different surface acid sites for 1,4-dioxane and H_2_O_2_ adsorption and reaction. Electron paramagnetic resonance measurements indicate that H_2_O_2_ forms hydroxyl radicals (˙OH) in the presence of CuO, and unusually, forms superoxide/peroxyl radicals (˙O_2_^−^) in the presence of WO_*x*_/ZrO_2_. The identified material properties suggest metal oxides/H_2_O_2_ as a potential advanced oxidation process in the treatment of 1,4-dioxane and other recalcitrant organic compounds.

## Abstract 2

FTIR and surface poisoning results suggest 1,4-dioxane adsorbs to Lewis acid sites on metal oxide surfaces and is degraded by surface radical species.

## Graphical abstract

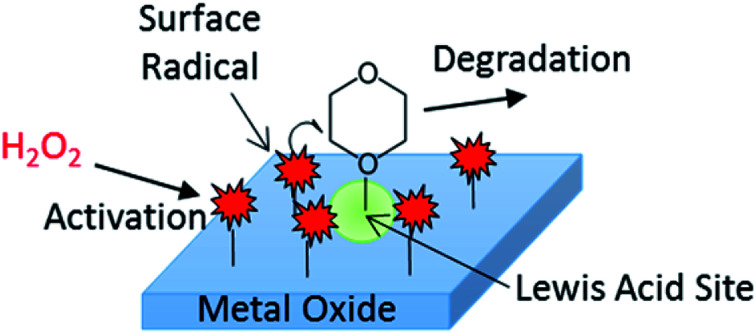

## 1. Introduction

1,4-dioxane is an industrial solvent,^1^ a byproduct present in personal-care products,^1^ and a suspected carcinogen with US state maximum contaminant limits that are frequently below 1 ppb. Because it is used to stabilize chlorinated volatile organic compound (CVOC) solvents,^1–5^ 1,4-dioxane was found by Adamson *et al.* to co-occur at 76% of California sites containing trichloroethane, and at 68% of sites containing 1,1-dichloroethene.^2^ Extrapolating this co-occurrence problem to the 15 000–25 000 US sites that are contaminated with CVOCs,^6^ 1,4-dioxane is likely widespread.

A number of advanced oxidation processes (AOPs) have been investigated for 1,4-dioxane remediation, including *ex situ* UV oxidative processes (UV/H_2_O_2_, UV/ozone),^7^ photocatalysis,^8–10^ or sonolysis and photocatalysis.^11^ A few studies looked at catalytic destruction of 1,4-dioxane, but most required the addition of high-energy resources such as UV light,^12,13^ ultrasonic waves,^14^ or electricity,^15–17^ which are not only costly from an energy perspective, but may also require the use of complicated reactor configurations and may not be suitable for universal treatment of 1,4-dioxane contamination (*e.g.* in chemical plumes).

“Dark” (non-photocatalytic) heterogeneous catalysis, which does not require additional energy sources, may be a more passive and more economically practical approach to treating 1,4-dioxane in water. Efforts have been made in the past to study the activation of chemical oxidants for AOP using materials as a heterogeneous form of Fenton's reagent, but few have focused much on 1,4-dioxane nor provided an experimental understanding of the surface chemistry.^18,19^ CuO has been investigated using ozone as oxidant, and implicated Lewis acid sites for the decomposition of ozone.^20^ A Pd-based catalyst was reported effective for degrading 1,4-dioxane using peroxymonosulfate, and suggested surface-bound radicals were responsible for degradation.^21^ Two studies discussed using H_2_O_2_; one a titanosilicate zeolite that was slightly active in water at 60 °C,^22^ and another concerning Fe(ii)-containing clays that showed degradation activity on the order of several days.^23^ Unexplored for 1,4-dioxane degradation and other contaminants are a number of materials reported to be able to nonphotocatalytically catalyze the dark dissociation of H_2_O_2_, such as ZrO_2_ ^24^ and TiO_2_.^25^ Furthermore, any materials properties of these unconventional H_2_O_2_-active materials which allow them to degrade organics is unclear.

In this study, we screened commercially available relatively inexpensive metal oxide H_2_O_2_-active catalysts to establish a set of basic data for the degradation of aqueous-phase 1,4-dioxane using H_2_O_2_ at mild ambient conditions and in the dark. A 1,4-dioxane degradation rate constant was measured for each material at room temperature and atmospheric pressure, and at near-neutral pH. H_2_O_2_ consumption activity (quantified as a rate constant) and consumption efficiency (quantified as moles of 1,4-dioxane degraded per mole H_2_O_2_ consumed) were also determined. To understand the essential surface properties that direct the degradation process, we further analyzed the materials using *in situ* Fourier-transform infrared (FTIR) analysis of acid sites using pyridine, acid site poisoning catalytic tests using acetonitrile, and electron paramagnetic resonance (EPR). Based on these results, we proposed a reaction mechanism to explain the observed material-dependency of 1,4-dioxane degradation.

## 2. Materials

CuO (>97%) and γ-Al_2_O_3_ (>97%) were used as received from Strem Chemicals. ZrO_2_ and TiO_2_ P25 were obtained from Evonik. Fe_2_O_3_ (hydrated, catalyst grade 30–50 mesh, crushed prior to characterization and kinetic experiments), WO_3_ (nanopowder), Zeolite Y (hydrogen, 30 : 1 SiO_2_ : Al_2_O_3_), CeO_2_ (99.95%, nanopowder), dichloromethane (chromasolv, 99.9%), and 1,4-dioxane (>99.5%), H_2_O_2_ (30%), and TiOSO_4_ (∼15 wt% in dilute sulfuric acid) were used as received from Sigma Aldrich. A zirconia-supported tungsten oxide material (“WO_*x*_/ZrO_2_”, 20 wt% WO_3_ content) was obtained from MEI Chemicals and used as-received. Specific surface areas (SSA) of the metal oxide materials were evaluated on a Quantachrome Autosorb IIIB using five-point BET calculations on samples degassed at 350 °C overnight. 5-Tert-butoxycarbonyl-5-methyl-1-pyrroline-*N*-oxide (BMPO) was obtained from Enzo Life Sciences. Deionized water was used in all experiments.

## 3. Analytical methods

### 3.1 Catalytic activity testing

For kinetic experiments, 171 mL of deionized (DI) water, 0.4 μL of 1,4-dioxane ([1,4-dioxane]_0_ = 27 μM, 2.3 ppm), and 0–600 μL of 30% H_2_O_2_ ([H_2_O_2_]_0_ = 0–30 mM) were added to a 250 mL Boston round bottle. After stirring, a ∼2 mL aliquot was taken from the reactor for baseline measurements of H_2_O_2_ and 1,4-dioxane. The catalyst was added to the reactor, which was then sealed with a septum, covered in foil to shield from ambient light, and magnetically stirred at 600 RPM. The amount of added catalyst was chosen such that the total exposed oxide surface area in the reaction medium was the same between experiments (475 m^2^ per L-fluid, Table S1†). Aliquots (∼1.5 mL) of reaction fluid were filtered with a 0.2 μm syringe filter to remove solid catalyst prior to H_2_O_2_ and 1,4-dioxane concentration measurements. Each reaction was repeated three times. No degradation was observed in experiments where only 1,4-dioxane and catalyst were present.

Because of order-of-magnitude differences in initial concentrations, the disappearance of H_2_O_2_ and 1,4-dioxane were both modeled as pseudo-first order processes, where the rate of disappearance of reactant X (either H_2_O_2_ or 1,4-dioxane), *r*_X_, is given by1
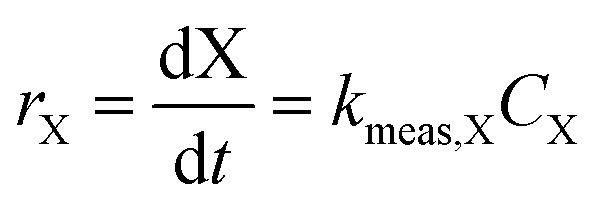
where *t* is time, *C*_X_ is the concentration of reactant X, and *k*_meas,X_ is the measured pseudo-first order rate constant, which can be found from integrating eqn (1) to obtain2
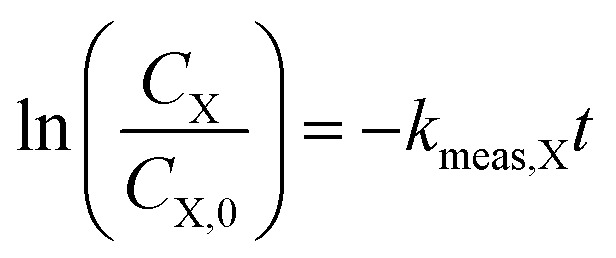
where *C*_X,0_ is the initial concentration of the reactant.

In order to better compare intrinsic catalytic activity, we report the pseudo-first order rate constant normalized by added catalyst surface area for each reagent for each catalyst, *k*_X_, since only exposed catalyst sites should be active. *k*_X_ is given by3
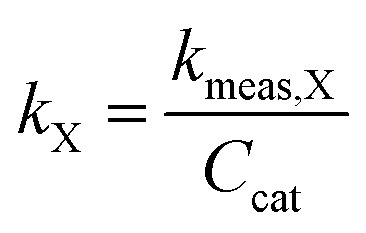
where *C*_cat_ is the concentration of catalyst surface area in the reactor (units of m^2^ mL^−1^), given by eqn (4)4
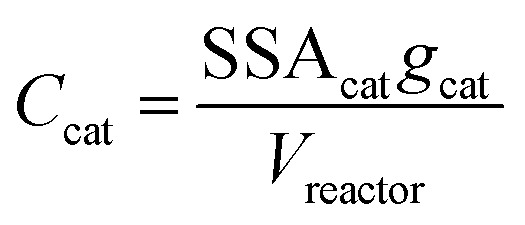
where SSA_cat_ is the specific surface area of the catalyst (units m^2^ g^−1^, as determined by BET, see Table S1†), *g*_cat_ is the grams catalyst added to the reactor (Table S1†), and *V*_reactor_ is the liquid reaction volume.

Selective site poisoning experiments were conducted using acetonitrile (which can chemisorb onto Lewis acid sites^26^), in which the catalysts were premixed with 318 μL of 0.1 M acetonitrile stock solution (final [acetonitrile] in reactor = 186 μM).^27^ Residual activity is defined as5
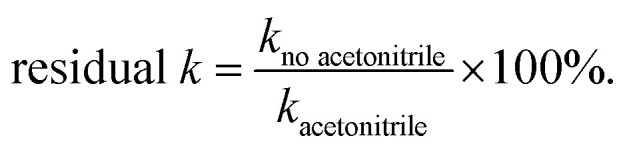


### 3.2 H_2_O_2_ quantification

H_2_O_2_ remaining in the aliquots was quantified using titanium oxysulfate (TiOSO_4_, ∼15 wt% in dilute H_2_SO_4_). TiOSO_4_ reacts with H_2_O_2_ to form the yellow-colored product perititanic acid, quantifiable using UV-vis.^28^ A standard curve was made using 0–80 μM H_2_O_2_ to verify that the response was linear at the maximum extinction (at *λ* = 405 nm). Reaction samples were diluted up to 20× to verify that absorbance at *λ* = 405 nm was in the standard.

### 3.3 1,4-Dioxane quantification

The concentration of 1,4-dioxane was determined as detailed by Li *et al.*^29^ Briefly, 0.5 mL of reaction aliquot and 0.5 mL of dichloromethane (DCM) was added to an autosampler vial and vigorously shaked for at least 30 seconds to extract the 1,4-dioxane into the DCM phase. Following freezing at −20 °C for at least 1 hour, the liquid DCM was decanted from the vial and put into a fresh autosampler vial containing ∼10 mg of sodium sulfate to sequester any residual water. These samples were then analyzed *via* GC-MS. A calibration curve was prepared using this method with 0–3200 ppb 1,4-dioxane in deionized water.

### 3.4 Determination of mass transfer resistances

To ensure the reported rate constants were kinetically limited, we evaluated external mass transfer resistances as we have done previously.^30,31^ We note that in this system, there is no gas phase reactants, therefore *k*_gl_ was neglected. Fig. S1,† prepared using CuO, one of the most active catalysts for H_2_O_2_ degradation, shows a linear increase in the measured rate constant with added catalyst, indicating there were no external mass transfer limitations in this range. The circled point corresponds to the SSA loading chosen for all other reactions.

To assess any internal mass transfer resistances, we determined the Weisz–Prater criterion (*C*_WP_) for both the first-order degradation of H_2_O_2_ and 1,4-dioxane according to^32,33^6
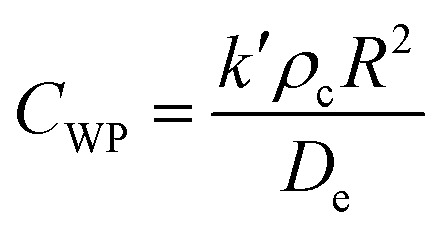
where *k*′ is the catalyst mass normalized rate constant (m^3^ s^−1^ g_cat_^−1^), *ρ*_c_ is the catalyst density (g m^−3^), *R* is the pellet radius (m, conservatively assumed to be 1 mm for all catalysts), and *D*_e_ is the effective diffusivity of H_2_O_2_ and 1,4-dioxane in the pores (assumed to be equivalent to H_2_O_2_ and 1,4-dioxane diffusivity in water, 5.0 × 10^−6^ m^2^ h^−1^ and 6.0 × 10^−8^ m^2^ h^−1^ respectively). As shown in Table S2,† the Weisz–Prater criterion was less than one for all materials, which implies that the catalysts' activities were not limited by internal diffusion.

### 3.5 EPR experiments

For the spin-trap experiments, 4 mM of CuO, WO_*x*_/ZrO_2_, or ZrO_2_ was added to a s 83 mM H_2_O_2_ and 5–10 mM BMPO solution, then mixed for ∼2 min. Transient radical species rapidly react with BMPO, forming stable adducts.^34–37^ 15 μL of the reaction mixture was sampled and sealed with Critoseal. EPR spectra of the spin trapped radicals were obtained using a Bruker EMX spectrometer at room temperature. EPR measurements were taken using a frequency of 9.30 GHz, power of 20 mW, modulation frequency of 100 kHz, modulation amplitude of 0.1 G, and time constant of 0.33 s. No radicals were detected in control solutions without metal oxide.

Freeze trapping was also attempted for direct detection of radicals. In these experiments, the 5 mm o.d. EPR tubes were rapidly frozen in an EtOH/dry ice bath before transferring into liquid N_2_. The measurements were conducted at 115 K using a frequency of 9.28 GHz; power of 1 mW; modulation frequency of 100 kHz, modulation amplitude of 2G, and time constant of 0.33 s.

### 3.6 Pyridine-FTIR

For the pyridine-FTIR experiments, the sample was pressed into a 13 mm diameter disk and analyzed in a homemade cell attached to a closed circulation system. Before pyridine adsorption, the cell was heated to 150 °C for 30 min at low pressure (<10^−3^ Pa) then allowed to cool to 25 °C. A spectrum was recorded as the background using a Bruker Tensor 27 FTIR spectrometer in transmittance mode. Gas-phase pyridine was then contacted with the sample for ∼20 min, and then the chamber evacuated at 150 °C for 30 min at low pressure (<1.0 × 10^−3^ Pa) to remove pyridine that was physically adsorbed before a FTIR spectrum of the chemisorbed pyridine was collected. Spectra were also taken after the sample was cooled to 25 °C.

For experiments looking at both pyridine and water, gas-phase water (∼1 atm) was added to the cell after the pyridine step. The water atmosphere was maintained for ∼20 min at 25 °C and then desorbed at 25 °C and 150 °C. The sample was brought to 25 °C, and an additional spectrum collected.

The Lewis acid site concentration is given by^38^7
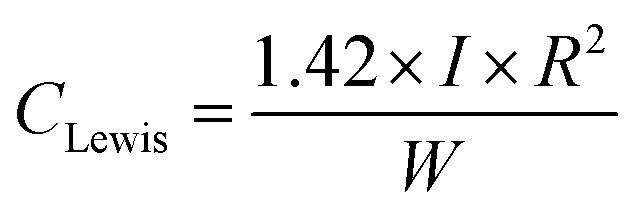
where *C*_Lewis_ is Lewis acid site concentration (mmol g_catalyst_^−1^), *R* is the catalyst radius (cm), *I* is the integration of the Lewis band (cm^−1^), *W* is the weight of disk (mg), and the factor 1.42 mol cm^−1^ is the extinction coefficient of pyridine adsorbed to an acid site.^38^ The *W* used for the experiments for CuO, ZrO_2_, and WO_*x*_/ZrO_2_ were 4.17, 27.2, and 39.5 mg respectively, and the *I* were found to be 1.095, 4.173, and 17.317 cm^−1^ respectively.

## 4. Results and discussion

**Fig. 1:**
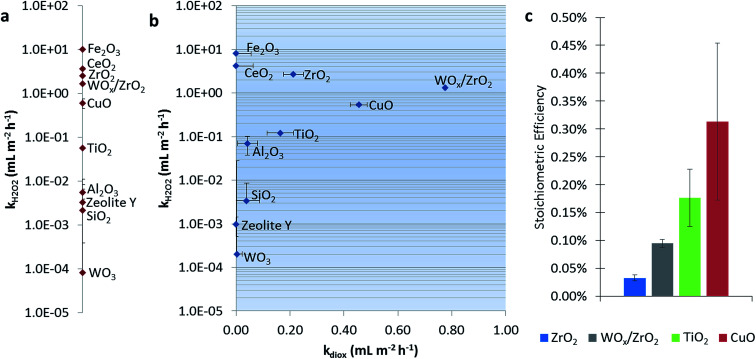
Pseudo-first order rate constants normalized by catalyst surface area for H_2_O_2_ consumption ([H_2_O_2_]_0_ = 15 mM, pH = 6.5) in the (a) absence and (b) presence of 1,4-dioxane ([1,4-dioxane]_0_ = 27 μM). Panel (b) shows the rate constant for H_2_O_2_ consumption plotted against the 1,4-dioxane degradation rate constant. (c) Stoichiometric efficiency (defined as moles 1,4-dioxane degraded per mole H_2_O_2_ consumed) calculated for catalysts active for 1,4-dioxane degradation at 10–12% conversion.

Fig. 1a shows the dark catalytic activities for H_2_O_2_ consumption alone (without 1,4-dioxane). CeO_2_, Fe_2_O_3_, and CuO all have high reaction rate constants, which generally agree with reported values of these Fenton-like materials, *i.e.*, those that activate H_2_O_2_*via* formal oxidation/reduction of the metal sites.^25,39^ The exception is CuO, which was slower than other reports (Table S3†), which may be due to differences in catalyst and H_2_O_2_ concentrations between studies. ZrO_2_ and TiO_2_ showed H_2_O_2_ degradation ability, in agreement with previous reports.^24,25,39–41^ SiO_2_ and Al_2_O_3_ exhibited only trace activity (also in agreement with literature),^25^ as was the case with zeolite Y. Monometallic WO_3_ was also nearly inactive.

Fig. 1b shows catalytic activities for the H_2_O_2_ consumption in the presence of 1,4-dioxane, and catalytic activity for 1,4-dioxane degradation. We denote the “best” 1,4-dioxane degradation catalysts as those that quickly degrade 1,4-dioxane (high *k*_diox_) while consuming low amounts of H_2_O_2_ (low *k*_H_2_O_2__), *i.e.*, those closest to the lower right hand side of the semi-log *k*-by-*k* plot. Catalytic activities for H_2_O_2_ consumption did not generally change in the presence of 1,4-dioxane (comparing Fig. 1a and b). Materials weakly active for H_2_O_2_ consumption (Al_2_O_3_, SiO_2_, and WO_3_) consumed H_2_O_2_ more rapidly, except for zeolite Y, which became less active; all these showed minimal 1,4-dioxane degradation ability.

Catalysts most active for H_2_O_2_ consumption were not the most effective at degrading 1,4-dioxane; Fe_2_O_3_ and CeO_2_, the catalysts with the highest *k*_H_2_O_2__, were inactive for 1,4-dioxane, and could be due to the conversion of H_2_O_2_ to nonreactive species under these reaction conditions (neutral pH). Materials that had the highest *k*_diox_ values were (listed in order of decreasing activity) WO_*x*_/ZrO_2_ ≫ CuO > ZrO_2_ ≫ TiO_2_. Their H_2_O_2_ consumption activity was also less than those of Fe_2_O_3_ and CeO_2_.

We calculated the stoichiometric efficiency for the four catalysts most active for 1,4-dioxane degradation during the batch reactions (Fig. 1c). Under these conditions, CuO was the most H_2_O_2_-efficient, followed by TiO_2_, WO_*x*_/ZrO_2_, and ZrO_2_. These values are on the same order of magnitude as those measured by Sedlak and co-workers for the degradation of phenol using H_2_O_2_ over a silica–Fe catalyst at neutral conditions (∼0.20–0.30%).^42,43^ A direct comparison of these results to other materials is made with caution, as the initial reactant amount and reactant type (50–250 mM H_2_O_2_ and 0.5 mM phenol *vs.* 15 mM H_2_O_2_ and 27 μM 1,4-dioxane) differ.

To further understand the surface mechanism, we explored the effects of initial H_2_O_2_ concentrations (1.5–30 mM) using a Langmuir–Hinshelwood–Hougen–Watson (LHHW) bimolecular surface reaction model, which assumes the two reactants (H_2_O_2_ and 1,4-dioxane) compete to adsorb to the same catalytic sites. The rate of H_2_O_2_ consumption (r′_H_2_O_2__) increased before plateauing at high [H_2_O_2_]_0_ for WO_*x*_/ZrO_2_, CuO, and ZrO_2_, which was indicative of saturation coverage of the active sites at high H_2_O_2_ concentrations (Fig. S2a†). The 1,4-dioxane degradation rate (*r*′_1,4-dioxane_), however, also increased and remained pseudo-first order with respect to [H_2_O_2_] (Fig. S2b†), which suggests that 1,4-dioxane adsorption (and subsequent reaction) sites are not blocked at high H_2_O_2_ concentrations, and implies that the adsorption and reaction occurs on sites different from those for H_2_O_2_ adsorption/reaction (for WO_*x*_/ZrO_2_, CuO, and ZrO_2_).

**Fig. 2:**
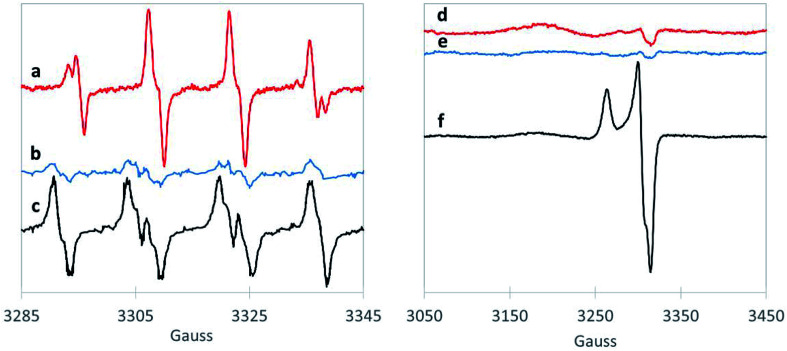
BMPO-trapping of radical species in the H_2_O_2_ consumption with (a) CuO (red), (b) ZrO_2_ (blue) and (c) WO_*x*_/ZrO_2_ (black). Spectra following freeze trapping over (d) CuO (red), (e) ZrO_2_ (blue) and (f) WO_*x*_/ZrO_2_ (black). The small features at 3320 G for traces (d) and (e) are artifacts from the EPR sample cavity.

We also examined the radicals formed from H_2_O_2_ activation by the 1,4-dioxane-active catalysts. Although EPR does not necessarily provide direct evidence of surface generation of radicals, it can reveal what radicals are generated in solution with the use of the spin-trap reagent. BMPO, the spin-trap reagent used here, rapidly reacts with otherwise transient radicals to form stable radical adducts with ˙OH and ˙O_2_^−^ species. The hyperfine structures of the BMPO adduct are characteristic of those of BMPO/˙OH (Fig. 2a), which fit well with two conformers with similar hyperfine splittings due to the nitrogen, the β hydrogen, and one of the γ hydrogen atoms,^35,44^ indicating formation of ˙OH radical over CuO. The EPR signal did not change with the addition of superoxide dismutase (“SOD,” an enzyme which rapidly and selectively converts ˙O_2_^−^ to O_2_ or H_2_O_2_), confirming that CuO did not generate ˙O_2_^−^ (Fig. S3†). This is in contrast to a recent study using ozone to degrade 1,4-dioxane over CuO, which formed primarily superoxide radical.^20^ However, as hydroxyl radical is amongst the strongest oxidants (Table S4†), it is likely responsible for the high 1,4-dioxane degradation ability of CuO.

The spectra of BMPO-radical adducts were significantly different when ZrO_2_ catalysts were exposed to H_2_O_2_ (Fig. 2b). The hyperfine structures of the EPR spectrum (Fig. 2b) indicates the formation of BMPO/˙O_2_^−^.^35^ BMPO/˙O_2_^−^ adduct was also observed in the reaction of WO_*x*_/ZrO_2_ (Fig. 2c) and the spectrum fit well with two conformers with similar hyperfine splittings due to the nitrogen and the β hydrogen atoms (Fig. S4a and b†). Moreover, addition of SOD significantly decreased the amount of BMPO-trapped radical (Fig. S3†), corroborating that superoxide is the primary radical generated by ZrO_2_ from H_2_O_2_. This observation is consistent with a previous work.^45^

Superoxide radicals have a lower oxidation potential compared to H_2_O_2_ ^46,47^ (Table S3†), and are considered relatively unreactive.^48^ It is surprising that this species (and not ˙OH) is generated by WO_*x*_/ZrO_2_, the most active catalyst tested for 1,4-dioxane degradation. This suggests some catalytically beneficial feature of ZrO_2_-supported WO_*x*_ domains that is absent from ZrO_2_ and from WO_3_. Direct freeze-trapping EPR measurements, in which H_2_O_2_/metal oxide suspensions are frozen and analyzed for any generated radicals (without using a spin-trap reagent), showed signals for superoxide radicals for WO_*x*_/ZrO_2_ (Fig. 2f). The *g*_x/y_ = 2.007 (3305 G) and *g*_z_ = 2.088 (broad peak centered at 3177 G) are typical of superoxide anion radicals in solution. The narrow peak at 3263 G (*g*_z_ = 2.032) and trough at 3315 G (*g*_xy_ = 2.002) are likely due to a WO_*x*_/ZrO_2_ surface-bound peroxyl radical whose g values, particularly *g*_z_, are perturbed due to its binding to the surface of the catalyst and much less spin–orbit coupling than that of free superoxide radical.^49^ The EPR spectrum can be fit well with a combination of these two types of radicals (Fig. S4c†). No signals were detected in freeze-trapping of ZrO_2_ and H_2_O_2_ even though ˙O_2_^−^ was spin-trapped using BMPO in the same reaction, suggesting that WO_*x*_/ZrO_2_ can better stabilize ˙O_2_^−^ radicals compared to ZrO_2_. In the CuO reaction with H_2_O_2_, the formation of ˙OH was too transient to be directly freeze-trapped.

Recognizing that 1,4-dioxane was historically used as a CVOC stabilizer due to its ability to complex with AlCl_3_ formed from CVOC storage inside aluminum-lined containers,^50,51^ we hypothesized that Lewis acid sites of the metal oxides are important for 1,4-dioxane degradation. We performed FTIR analysis of 1,4-dioxane-active materials using pyridine as probe molecule for surface acid sites under dry as well as humid conditions to simulate the aqueous-phase conditions of the oxidation reaction.

**Fig. 3:**
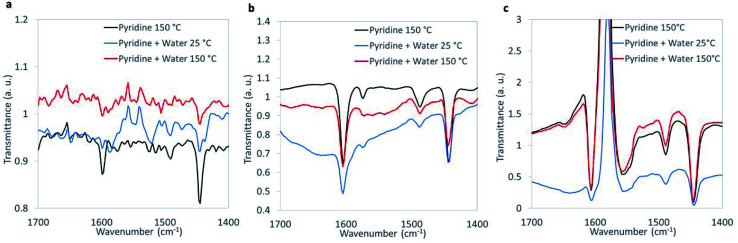
FTIR spectra of chemisorbed pyridine under dehydrated and humid conditions for (a) CuO, (b) ZrO_2_, and (c) WO_*x*_/ZrO_2_.

Characteristic peaks at ∼1445 cm^−1^ assigned to pyridine adsorbed on Lewis acid sites were observed on WO_*x*_/ZrO_2_, ZrO_2_, and CuO (Fig. 3). The concentration of Lewis acid sites was quantified by integrating the area of the 1445 cm^−1^ peak, then normalizing by the amount of catalytic material and using an extinction coefficient previously determined by Emeis^38^ (Table S5†). The metal oxides had similar Lewis acid site densities, which were lower than the theoretical metal site density of ∼4 atoms per nm^−2^ (roughly 15–20%).^52–54^

**Fig. 4:**
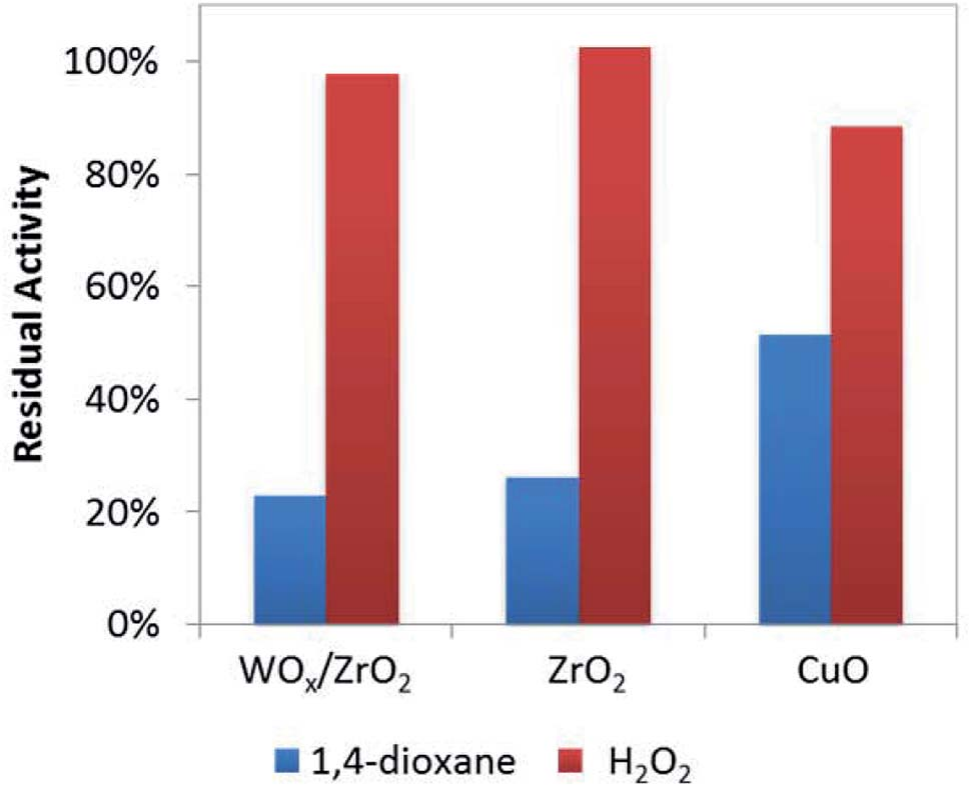
Residual activity of catalysts for 1,4-dioxane and H_2_O_2_ degradation with the addition of 0.125 moles acetonitrile per mole surface site (186 μM acetonitrile).

To help verify these Lewis acid sites participate in 1,4-dioxane oxidation catalysis, we co-added acetonitrile to the batch reactor tests (at amount equivalent to ∼12.5% of theoretical metal site density, Fig. 4) and quantified the resulting rate constants. As a water-soluble Lewis base (less basic but easier to handle compared to pyridine), acetonitrile lowered 1,4-dioxane degradation substantially (by ∼75–80%) over ZrO_2_ and WO_*x*_/ZrO_2_, but did not affect H_2_O_2_ consumption much (by <5%) (Fig. 4). The Lewis acid sites of ZrO_2_ and WO_*x*_/ZrO_2_ are the likely adsorption sites for 1,4-dioxane (as poisoned by acetonitrile). Acetonitrile inhibited 1,4-dioxane degradation over CuO to a lesser extent (∼48%) and H_2_O_2_ consumption to a greater extent (∼13%).

Humidification, which more closely resembles the aqueous reaction conditions, was introduced during FTIR analysis (Fig. 3). The introduction of water led to the appearance of Brønsted peaks (identified by characteristic pyridine IR peak at ∼1550 cm^−1^) on CuO but not ZrO_2_ or WO_*x*_/ZrO_2_. We suggest that CuO, when in water, contains Brønsted acid sites that may contribute 1,4-dioxane degradation, and that Brønsted acidity may not be an important characteristic for ZrO_2_ or WO_*x*_/ZrO_2_ catalysis.

**Scheme 1:**
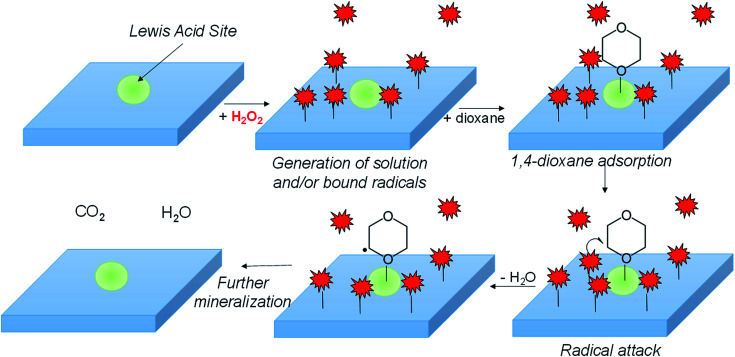
Hypothesized general mechanism of 1,4-dioxane degradation over Lewis-acidic catalyst surfaces. 1,4-dioxane adsorbed to Lewis acid sites reacts with radicals generated from activation of H_2_O_2_ on surface.

In combining the kinetic, EPR, FTIR, and surface poisoning results, we propose H_2_O_2_ dissociates onto metal surface sites into either surface adsorbed ˙OH or ˙O_2_^−^ over the metal oxide surface, while Lewis-acidic sites (a minority of total sites) adsorb 1,4-dioxane (Scheme 1). The adsorbed radicals, or peroxyl radicals, react with adsorbed 1,4-dioxane, which contributes to more efficient use of H_2_O_2_ and to higher 1,4-dioxane degradation activity.

## 5. Conclusions

In this work, we surveyed a number of H_2_O_2_-active metal oxide materials, and evaluated their efficacy for the aqueous catalytic degradation of 1,4-dioxane at mild conditions (ambient temperature, neutral pH, in the dark). Of the materials screened, we found that WO_*x*_/ZrO_2_ had the highest 1,4-dioxane degradation rate, followed by CuO and ZrO_2_. A LHHW analysis indicated that H_2_O_2_ and 1,4-dioxane adsorb to distinct catalytic sites. EPR measurements indicate that CuO activates H_2_O_2_ into highly reactive hydroxyl radicals, while ZrO_2_ and WO_*x*_/ZrO_2_ form solely less-active superoxide radicals. Pyridine-FTIR and selective poisoning experiments imply that 1,4-dioxane, a weak Lewis base, selectively adsorbs to Lewis acid sites on the catalysts. We present a possible surface-reaction mechanism in which 1,4-dioxane adsorbs to catalyst sites and reacts with metal-oxide supported radical species. These materials show promise for treatment of 1,4-dioxane contaminated waters.

## Conflicts of interest

There are no conflicts to declare.

## Supplementary Material

RA-009-C9RA05007H-s001

## References

[cit1] Mohr T. K. , 1,4-Dioxane and other Solvent Stabilizers, San Jose, CA, June 14, 2001

[cit2] Adamson D. T., Mahendra S., Walker K. L., Rauch S. R., Sengupta S., Newell C. J. (2014). A Multisite Survey To Identify the Scale of the 1,4-Dioxane Problem at Contaminated Groundwater Sites. Environ. Sci. Technol. Lett..

[cit3] Anderson R. H., Anderson J. K., Bower P. A. (2012). Co-occurrence of 1,4-dioxane with trichloroethylene in chlorinated solvent groundwater plumes at US Air Force installations: Fact or fiction. Integr. Environ. Assess. Manage..

[cit4] Zenker M. J., Borden R. C., Barlaz M. A. (2003). Occurrence and treatment of 1,4-dioxane in aqueous environments. Environ. Eng. Sci..

[cit5] Mohr T. , Stickney J. and Diguiseppi W., Environmental investigation and remediation: 1, 4-dioxane and other solvent stabilizers, Environmental investigation and remediation: 1, 4-dioxane and other solvent stabilizers, 2010

[cit6] Sale T. , Newell C., Stroo H., Hinchee R. and Johnson P., Regarding Management of Chlorinated Solvents in Soils and Groundwater, 2008

[cit7] Otto M., Nagaraja S. (2007). Treatment technologies for 1,4-dioxane: Fundamentals and field applications. Remediat. J..

[cit8] Lam S. W., Hermawan M., Coleman H. M., Fisher K., Amal R. (2007). The role of copper(ii) ions in the photocatalytic oxidation of 1,4-dioxane. J. Mol. Catal. A: Chem..

[cit9] Vescovi T., Coleman H. M., Amal R. (2010). The effect of pH on UV-based advanced oxidation technologies – 1,4-Dioxane degradation. J. Hazard. Mater..

[cit10] Barndõk H., Hermosilla D., Han C., Dionysiou D. D., Negro C., Blanco Á. (2016). Degradation of 1,4-dioxane from industrial wastewater by solar photocatalysis using immobilized NF-TiO_2_ composite with monodisperse TiO_2_ nanoparticles. Appl. Catal., B.

[cit11] Nakajima A., Tanaka M., Kameshima Y., Okada K. (2004). Sonophotocatalytic destruction of 1,4-dioxane in aqueous systems by HF-treated TiO_2_ powder. J. Photochem. Photobiol., A.

[cit12] Son H.-S., Im J.-K., Zoh K.-D. (2009). A Fenton-like degradation mechanism for 1,4-dioxane using zero-valent iron (Fe0) and UV light. Water Res..

[cit13] Barndõk H., Blanco L., Hermosilla D., Blanco Á. (2016). Heterogeneous photo-Fenton processes using zero valent iron microspheres for the treatment of wastewaters contaminated with 1,4-dioxane. Chem. Eng. J..

[cit14] Beckett M. A., Hua I. (2003). Enhanced sonochemical decomposition of 1,4-dioxane by ferrous iron. Water Res..

[cit15] Jasmann J. R., Borch T., Sale T. C., Blotevogel J. (2016). Advanced Electrochemical Oxidation of 1,4-Dioxane via Dark Catalysis by Novel Titanium Dioxide (TiO_2_) Pellets. Environ. Sci. Technol..

[cit16] De Clercq J., Van de Steene E., Verbeken K., Verhaege M. (2010). Electrochemical oxidation of 1, 4-dioxane at boron-doped diamond electrode. J. Chem. Technol. Biotechnol..

[cit17] Choi J. Y., Lee Y.-J., Shin J., Yang J.-W. (2010). Anodic oxidation of 1, 4-dioxane on boron-doped diamond electrodes for wastewater treatment. J. Hazard. Mater..

[cit18] Bokare A. D., Choi W. (2014). Review of iron-free Fenton-like systems for activating H_2_O_2_ in advanced oxidation processes. J. Hazard. Mater..

[cit19] Wu S., He H., Li X., Yang C., Zeng G., Wu B., He S., Lu L. (2018). Insights into atrazine degradation by persulfate activation using composite of nanoscale zero-valent iron and graphene: Performances and mechanisms. Chem. Eng. J..

[cit20] Basso A., Landers R., Alvarez P. J. J., Puma G. L., Moreira R. F. P. M. (2018). Treatment of aqueous solutions of 1,4-dioxane by ozonation and catalytic ozonation with copper oxide (CuO) AU – Scaratti, Gidiane. Environ. Technol..

[cit21] Feng Y., Lee P.-H., Wu D., Shih K. (2017). Surface-bound sulfate radical-dominated degradation of 1,4-dioxane by alumina-supported palladium (Pd/Al_2_O_3_) catalyzed peroxymonosulfate. Water Res..

[cit22] Fan W., Kubota Y., Tatsumi T. (2008). Oxidation of 1,4-Dioxane over Ti-MWW in the Presence of H_2_O_2_. ChemSusChem.

[cit23] Zeng Q., Dong H., Wang X., Yu T., Cui W. (2017). Degradation of 1, 4-dioxane by hydroxyl radicals produced from clay minerals. J. Hazard. Mater..

[cit24] Lousada C. M., Jonsson M. (2010). Kinetics, Mechanism, and Activation Energy of H_2_O_2_ Decomposition on the Surface of ZrO_2_. J. Phys. Chem. C.

[cit25] Hiroki A., LaVerne J. A. (2005). Decomposition of Hydrogen Peroxide at Water–Ceramic Oxide Interfaces. J. Phys. Chem. B.

[cit26] Harris J. W., Cordon M. J., Di Iorio J. R., Vega-Vila J. C., Ribeiro F. H., Gounder R. (2016). Titration and quantification of open and closed Lewis acid sites in Sn-Beta zeolites that catalyze glucose isomerization. J. Catal..

[cit27] Wachs I. E. , Brundle C. R. and Evans J. C. A., Characterization of Catalytic Materials, 2009

[cit28] Satterfield C. N., Bonnell A. H. (1955). Interferences in Titanium Sulfate Method for Hydrogen Peroxide. Anal. Chem..

[cit29] Li M., Conlon P., Fiorenza S., Vitale R. J., Alvarez P. J. J. (2011). Rapid Analysis of 1,4-Dioxane in Groundwater by Frozen Micro-Extraction with Gas Chromatography/Mass Spectrometry. Groundwater Monit. Rem..

[cit30] Fang Y.-L., Heck K. N., Alvarez P. J. J., Wong M. S. (2011). Kinetics Analysis of Palladium/Gold Nanoparticles as Colloidal Hydrodechlorination Catalysts. ACS Catal..

[cit31] Qian H., Zhao Z., Velazquez J. C., Pretzer L. A., Heck K. N., Wong M. S. (2014). Supporting palladium metal on gold nanoparticles improves its catalysis for nitrite reduction. Nanoscale.

[cit32] Weisz P. B. and Prater C. D., Interpretation of Measurements in Experimental Catalysis, in Advances in Catalysis, ed. W. G. Frankenburg, V. I. Komarewsky and E. K. Rideal, Academic Press, 1954, vol. 6, pp. 143–196

[cit33] Soultanidis N., Zhou W., Psarras A. C., Gonzalez A. J., Iliopoulou E. F., Kiely C. J., Wachs I. E., Wong M. S. (2010). Relating *n*-Pentane Isomerization Activity to the Tungsten Surface Density of WO_*x*_/ZrO_2_. J. Am. Chem. Soc..

[cit34] Gopalakrishnan B., Nash K. M., Velayutham M., Villamena F. A. (2012). Detection of nitric oxide and superoxide radical anion by electron paramagnetic resonance spectroscopy from cells using spin traps. J. Visualized Exp..

[cit35] Zhao H., Joseph J., Zhang H., Karoui H., Kalyanaraman B. (2001). Synthesis and biochemical applications of a solid cyclic nitrone spin trap: a relatively superior trap for detecting superoxide anions and glutathiyl radicals. Free Radical Biol. Med..

[cit36] Shi H., Timmins G., Monske M., Burdick A., Kalyanaraman B., Liu Y., Clément J.-L., Burchiel S., Liu K. J. (2005). Evaluation of spin trapping agents and trapping conditions for detection of cell-generated reactive oxygen species. Arch. Biochem. Biophys..

[cit37] Liu D., Xiu Z., Liu F., Wu G., Adamson D., Newell C., Vikesland P., Tsai A.-L., Alvarez P. J. (2013). Perfluorooctanoic acid degradation in the presence of Fe(iii) under natural sunlight. J. Hazard. Mater..

[cit38] Emeis C. A. (1993). Determination of Integrated Molar Extinction Coefficients for Infrared Absorption Bands of Pyridine Adsorbed on Solid Acid Catalysts. J. Catal..

[cit39] Lousada C. M., Yang M., Nilsson K., Jonsson M. (2013). Catalytic decomposition of hydrogen peroxide on transition metal and lanthanide oxides. J. Mol. Catal. A: Chem..

[cit40] Lousada C. M., Johansson A. J., Brinck T., Jonsson M. (2012). Mechanism of H_2_O_2_ Decomposition on Transition Metal Oxide Surfaces. J. Phys. Chem. C.

[cit41] Lousada C. M., Johansson A. J., Brinck T., Jonsson M. (2013). Reactivity of metal oxide clusters with hydrogen peroxide and water – a DFT study evaluating the performance of different exchange-correlation functionals. Phys. Chem. Chem. Phys..

[cit42] Pham A. L.-T., Doyle F. M., Sedlak D. L. (2012). Kinetics and efficiency of H_2_O_2_ activation by iron-containing minerals and aquifer materials. Water Res..

[cit43] Pham A. L.-T., Lee C., Doyle F. M., Sedlak D. L. (2009). A Silica-Supported Iron Oxide Catalyst Capable of Activating Hydrogen Peroxide at Neutral pH Values. Environ. Sci. Technol..

[cit44] Baldrian P., Merhautová V., Gabriel J., Nerud F., Stopka P., Hrubý M., Beneš M. J. (2006). Decolorization of synthetic dyes by hydrogen peroxide with heterogeneous catalysis by mixed iron oxides. Appl. Catal., B.

[cit45] Giamello E., Rumori P., Geobaldo F., Fubini B., Paganini M. (1996). The interaction between hydrogen peroxide and metal oxides: EPR investigations. Appl. Magn. Reson..

[cit46] Bockris J. O. M., Oldfield L. F. (1955). The oxidation-reduction reactions of hydrogen peroxide at inert metal electrodes and mercury cathodes. Trans. Faraday Soc..

[cit47] Buettner G. R. (1993). The Pecking Order of Free Radicals and Antioxidants: Lipid Peroxidation, α-Tocopherol, and Ascorbate. Arch. Biochem. Biophys..

[cit48] Nordberg J., Arnér E. S. J. (2001). Reactive oxygen species, antioxidants, and the mammalian thioredoxin system1. Free Radical Biol. Med..

[cit49] Murphy D. M., Giamello E. (2002). EPR of Paramagnetic Centres on Solid Surfaces. Electron Paramagn. Reson..

[cit50] Archer W. L. (1984). A laboratory evaluation of 1,1,1-Trichloroethane-Metal-Inhibitor systems. Mater. Corros..

[cit51] Archer W. L. (1982). Aluminum-1, 1, 1-trichloroethane. Reactions and inhibition. Ind. Eng. Chem. Prod. Res. Dev..

[cit52] Kim T., Burrows A., Kiely C. J., Wachs I. E. (2007). Molecular/electronic structure–surface acidity relationships of model-supported tungsten oxide catalysts. J. Catal..

[cit53] Wachs I. E. (1996). Raman and IR studies of surface metal oxide species on oxide supports: Supported metal oxide catalysts. Catal. Today.

[cit54] Knowles W. V. , Nutt M. O. and Wong M. S., Supported metal oxides and the surface density metric, in Handbook of Catalyst Synthesis: The Science and Engineering of Catalyst Preparation, ed. Regalbuto J. R., Taylor & Francis, Boca Raton, FL, 2007, pp. 251–281

