# Supplementary material for: Effectiveness of metal oxide catalysts for the degradation of 1,4-dioxane
Source: RSC Adv. 2019 Aug 28;9(46):27042–9. doi: 10.1039/c9ra05007h (PMC9070425; doi:10.1039/c9ra05007h)
Supplement: RA-009-C9RA05007H-s001 [file RA-009-C9RA05007H-s001.pdf]

Electronic Supplementary Material (ESI) for RSC Advances.
This journal is © The Royal Society of Chemistry 2019

Supplementary Information for

Effectiveness of Metal Oxide Catalysts for the Dark Degradation of 1,4-Dioxane

Kimberly N. Heck,[1] Yehong Wang,[2] Gang Wu,[3] Feng Wang,[2] Ah-lim Tsai,[3] David T. Adamson,[4] Michael S. Wong[1,5,6,7,8*]

[1]Department of Chemical and Biomolecular Engineering, Rice University, Houston, TX, 77005, USA
[2]Dalian Institute of Chemical Physics, Chinese Academy of Sciences, Dalian, China
[3]Division of Hematology, Department of Internal Medicine, University of Texas-Medical School at Houston, Houston, TX, 77030, USA
[4]GSI Environmental, Houston, TX, 77098, USA
[5]Department of Chemistry, [6]Department of Civil and Environmental Engineering, [7]Department of Materials Science and NanoEngineering, [8]Center for Nano-Enabled Water Treatment, Rice University, Houston, TX, 77005, USA

Table S1. Catalyst surface areas and amounts charged to reactor

Catalyst	Surface Area (m^2 g^{-1})	Reactor Charge (g)
CuO	138	0.59
Fe_2O_3	146	0.55
TiO_2	65	1.25
ZrO_2	70.42	1.15
CeO_2	45.36	1.79
WO_3	7.28	11.16
WO_x/ZrO_2	275	0.30
H Zeolite Y	775	0.10
SiO_2	447.8	0.18
Al_2O_3	149.8	0.54

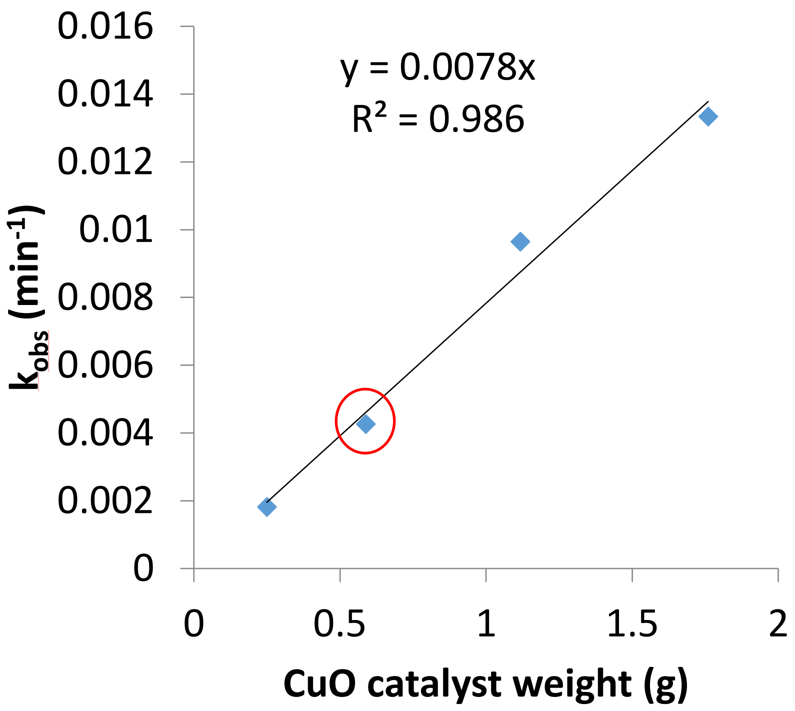

Figure S1. Determination of operating conditions to operate in kinetic regime (no mass transfer limitations) using CuO, one of the fastest catalysts for H_2O_2 degradation, shows a linear increase in the measured first order rate constant with added catalyst. The circled point corresponds to the SSA chosen for all other reactions.

Table S2. Weisz-Prater criterion of catalysts for H_2O_2 and 1,4-dioxane degradation

Catalyst	Amount added to reactor (g)	Solid density, ρ_c (kg m^{-3})	k'_{H2O2} (m^3 h^{-1} kg^{-1})	k'_{diox} (m^3 h^{-1} kg^{-1})	C_{WP} (H_2O_2)[a]	C_{WP} (1,4-dioxane)[a]
Fe_2O_3	0.56	3.4×10^{-6}	6882	0	4.5×10^{-3}	0
CuO	0.59	3.25×10^{-6}	441	374	3.0×10^{-4}	2.1×10^{-2}
CeO_2	1.79	1.1×10^{-6}	1116	0	2.3×10^{-3}	0
SiO_2	0.18	1.1×10^{-6}	8.81	105	1.9×10^{-6}	1.8×10^{-3}
Al_2O_3	0.54	3.2×10^{-6}	60	37	3.9×10^{-5}	1.9×10^{-3}
H^+-Zeolite Y	0.10	6.1×10^{-7}	0	0	0	0
TiO_2	1.25	7.3×10^{-6}	46.4	62.4	6.8×10^{-5}	7.6×10^{-3}
ZrO_2	1.15	6.7×10^{-6}	1109	0.1	1.5×10^{-3}	9.8×10^{-3}
WO_3	11.1	6.5×10^{-5}	0.51	0.098	6.7×10^{-6}	1.1×10^{-4}
20 wt% WO_x/ZrO_2	0.30	1.7×10^{-6}	2132	1252	7.3×10^{-4}	3.6×10^{-2}

[a]Calculated assuming a conservative pellet radius of 1 mm

Table S3. Comparison of k_{H2O2} with literature values

Material	This Work		From Ref[1]		From Ref [2-4]	
	C_{cat} (m_{cat}^2 mL^{-1})	k_{H2O2} (L m_{cat}^{-2} s^{-1})	C_{cat} (m_{cat}^2 mL^{-1})	k_{H2O2} (L m_{cat}^{-2} s^{-1})	C_{cat} (m_{cat}^2 mL^{-1})	k_{H2O2} (L m_{cat}^{-2} s^{-1})
SiO_2	0.475	5.97E-10	1.32	1.81E-09	-	-
Al_2O_3	0.475	1.54E-09	13.48	3.72E-09	-	-
TiO_2	0.475	1.58E-08	0.42	9.32E-07	-	-
ZrO_2	0.475	7.08E-07	0.27	4.92E-06	0.15	4.10E-07
CeO_2	0.475	1.02E-06	1.20	5.27E-07	0.15	1.13E-06
Fe_2O_3	0.475	2.80E-06	-	-	0.09	2.33E-06
CuO	0.475	1.66E-07	-	-	0.006	3.17E-05
H^+- Zeolite Y	0.475	9.00E-10	-	-	-	-
WO_3	0.475	2.25E-11	-	-	-	-
20 wt% WO_x/ZrO_2	0.475	4.59E-07	-	-	-	-

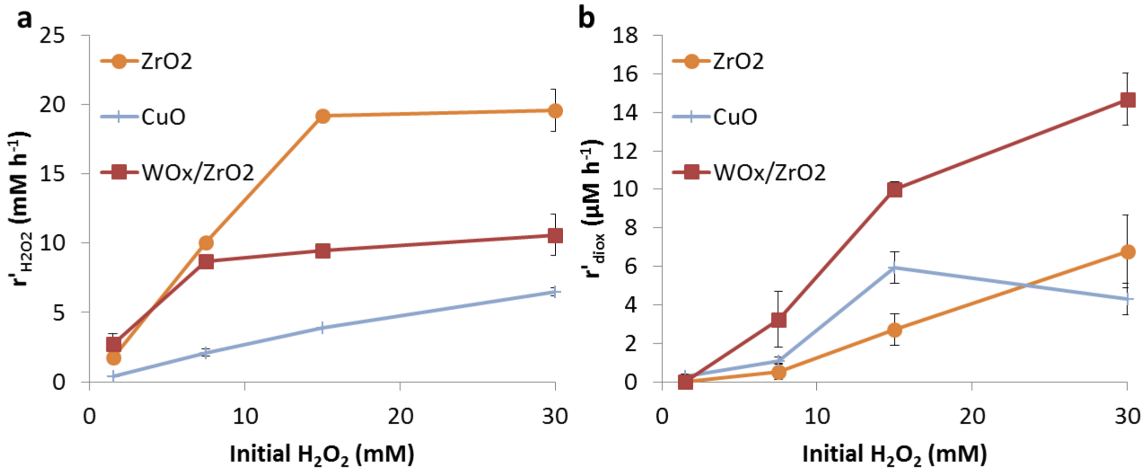

Figure S2. Effect of initial H_2O_2 on (a) r'_{H2O2}, and (b) r'_{diox}. [1,4-dioxane]$_0$ = 27 µM

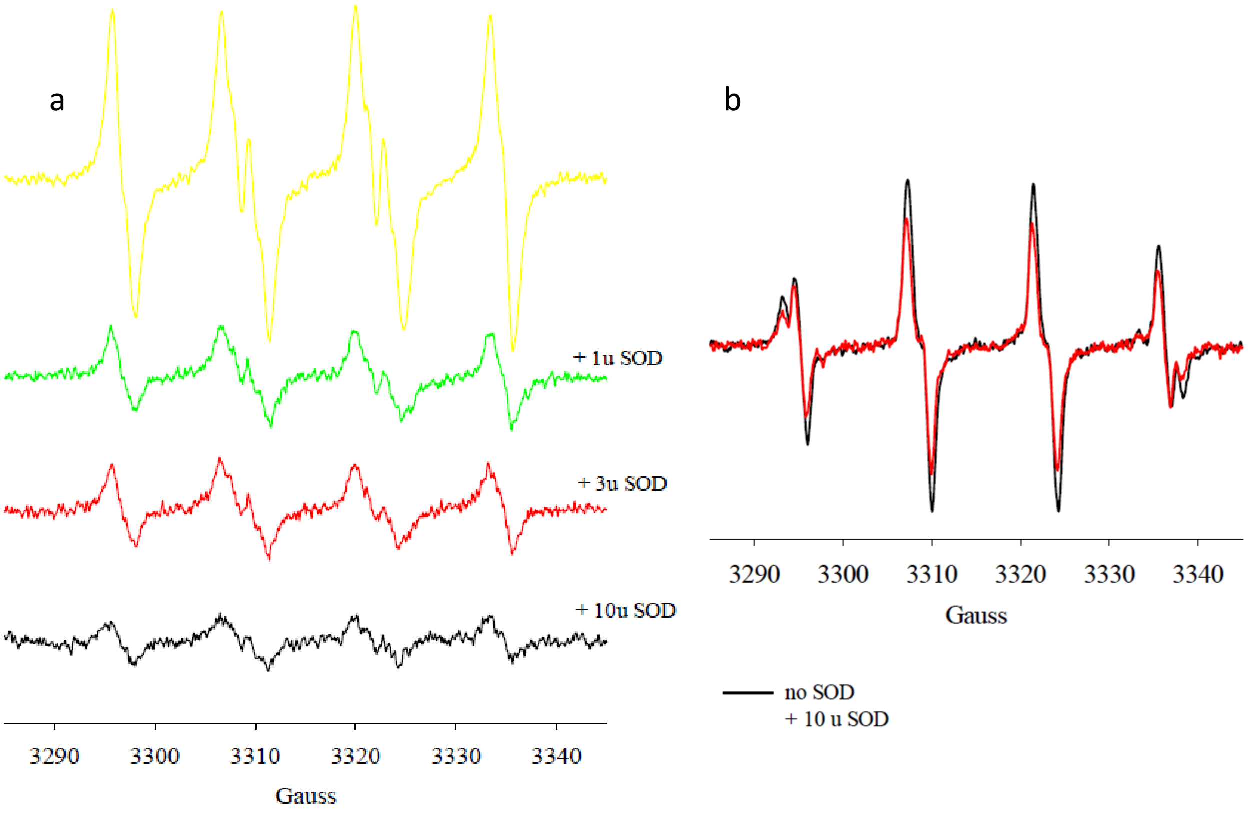

Figure S3. EPR signals for H_2O_2, BMPO and 20 wt% WO_x/ZrO_2 (a) or CuO (b) in the presence of SOD.

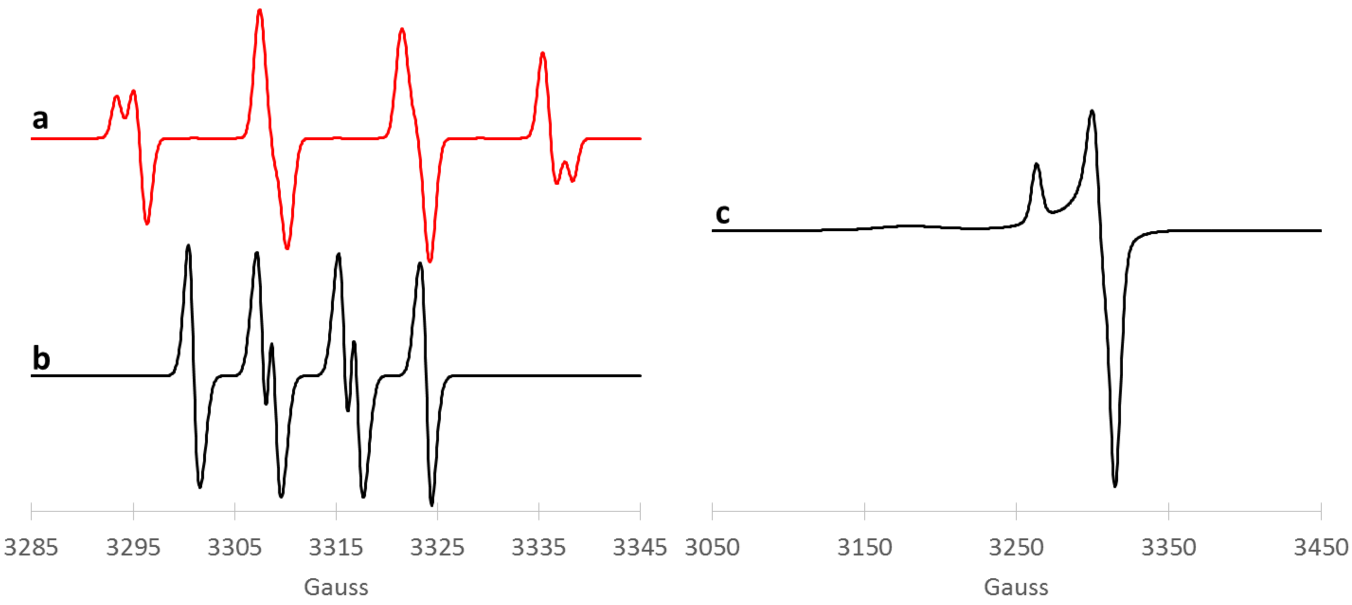

Figure S4. Simulated EPR spectra of (a) BMPO/•OH, (b) BMPO/•O_2^-, and (c) combined spectra of free and surface bound •O_2^- (or a superoxide and a peroxyl radical).

Table S4. Oxidation Potentials of selected oxidants at pH 7

Oxidant	E^0 (V)
•OH	2.31 [a]
H_2O_2	1.77 [b]
O_2•$^-$	1.06 [a]

[a]From reference[5]

[b]From reference[6]

Table S5. Lewis acid site densities of catalytic materials.

**Catalyst**	**Concentration of Lewis acid sites (mmol g_{cat}^{-1})**	**Lewis acid site density (atoms nm^{-2})**	**Surface metal sites that are Lewis acid sites[a]**
CuO	0.160	0.69	17.2%
ZrO_2	0.092	0.79	19.7%
WO_x/ZrO_2	0.260	0.58	14.4%

[a] Calculated assuming surface density of 4 atoms nm^{-2}, from ref [7,8]

SI References

1 Hiroki, A. & LaVerne, J. A. Decomposition of Hydrogen Peroxide at Water−Ceramic Oxide Interfaces. *The Journal of Physical Chemistry B* **109**, 3364-3370, doi:10.1021/jp046405d (2005).

2 Lousada, C. M., Yang, M., Nilsson, K. & Jonsson, M. Catalytic decomposition of hydrogen peroxide on transition metal and lanthanide oxides. *Journal of Molecular Catalysis A: Chemical* **379**, 178-184, doi:http://dx.doi.org/10.1016/j.molcata.2013.08.017 (2013).

3 Lousada, C. M. & Jonsson, M. Kinetics, Mechanism, and Activation Energy of H2O2 Decomposition on the Surface of ZrO2. *The Journal of Physical Chemistry C* **114**, 11202-11208, doi:10.1021/jp1028933 (2010).

4 Lousada, C. M., Johansson, A. J., Brinck, T. & Jonsson, M. Mechanism of H2O2 Decomposition on Transition Metal Oxide Surfaces. *The Journal of Physical Chemistry C* **116**, 9533-9543, doi:10.1021/jp300255h (2012).

5 Buettner, G. R. The Pecking Order of Free Radicals and Antioxidants: Lipid Peroxidation, α-Tocopherol, and Ascorbate. *Archives of Biochemistry and Biophysics* **300**, 535-543, doi:http://dx.doi.org/10.1006/abbi.1993.1074 (1993).

6 Bockris, J. O. M. & Oldfield, L. F. The oxidation-reduction reactions of hydrogen peroxide at inert metal electrodes and mercury cathodes. *Transactions of the Faraday Society* **51**, 249-259, doi:10.1039/TF9555100249 (1955).

7 Kim, T., Burrows, A., Kiely, C. J. & Wachs, I. E. Molecular/electronic structure–surface acidity relationships of model-supported tungsten oxide catalysts. *Journal of Catalysis* **246**, 370-381 (2007).

8 Wachs, I. E. Raman and IR studies of surface metal oxide species on oxide supports: Supported metal oxide catalysts. *Catalysis Today* **27**, 437-455, doi:http://dx.doi.org/10.1016/0920-5861(95)00203-0 (1996).
